# A scoping review on the impact of versatile Digital Health innovations on pharmacy education

**DOI:** 10.3389/fmed.2025.1577494

**Published:** 2025-10-17

**Authors:** Fahad T. Alsulami

**Affiliations:** Clinical Pharmacy Department, College of Pharmacy, Taif University, Taif, Saudi Arabia

**Keywords:** Digital Health, health literacy, curriculum integration, telehealth training, pharmacy education, telepharmacy, artificial intelligence

## Abstract

**Background:**

Digital Health innovative technologies, encompassing eHealth, mHealth, e-learning, tele-health, artificial intelligence (AI), tele-medicine, tele pharmacy, virtual reality, and augmented reality, are increasingly incorporated into learning and pharmacy education to prepare students for a Digital healthcare environment. However, evidence on the impact and implementation of these technologies still needs to be explored.

**Objective:**

The current scoping review collates and appraises the impact of Digital Health on pharmacy education, evaluating effects on learning outcomes, skill development, competencies, and readiness for tele pharmacy and Digital Health application transformations. The primary objective was to explore the impact of Digital Health (eHealth, mHealth, e learning, telehealth, AI, telemedicine, tele pharmacy, VR, ML, and AR) in pharmacy education via structured scoping review reporting.

**Methods:**

A systematic search following PRISMA guidelines conducted across databases, including Cochrane Library, Google Scholar, PubMed, Scopus, and Web of Science for recent papers published on Digital Health in pharmacy education from 2019 to 2024. Post-screening, 47 studies met the final criteria.

**Results:**

47 studies were included in the current scoping review. Five themes emerged (1) Curriculum integration and transformation in pharmacy education; (2) Digital literacy (competency development); (3) Tele pharmacy (remote health services and AI) in education; (4) Practical skill development (interactive learning through Digital tools) and (5) Student and faculty perceptions, attitudes, and challenges in adopting Digital Health. Digital Health integration improves students’ Digital competence, engagement, and tele pharmacy readiness, though gaps remain in curriculum standardization. Regional disparities show integration, which more advanced in Western programs, while foundational efforts seen in Asian, Middle East and North Africa (MENA) regions. The current review emphasized the importance of faculty developing, workload integration, regional disparities, and ethical concerns with AI and challenges in hybrid learning.

**Conclusion:**

Digital Health has been an innovator in pharmacy students’ education, equipping them with the skills and competences required in today’s healthcare environment. However, faculty development, curriculum gaps, workload integration, insufficient telehealth training, regional disparities, and inadequate AI ethics instruction all point to the need for adequate and relevant curriculum modifications to qualify graduates to deal with Digital healthcare challenges.

## Highlights

- Digital Health tools are increasingly integrated into pharmacy education to prepare students for modern healthcare.
- Many pharmacy programs lack formal Digital Health training, revealing a substantial gap in student readiness.
- Students show varied Digital literacy levels, pointing to a need for targeted training in AI and Digital tools.
- While students generally have a positive view of Digital Health, concerns about AI ethics, data security, and hands-on skill retention suggest the need for balanced training approach.

## 1 Background

Digital Health encompassing eHealth, mHealth, e learning, tele-health, artificial intelligence (AI), tele-medicine, tele pharmacy, Machine Learning (ML), virtual reality (VR), and augmented reality (AR), and electronic health records (EHRs). These technologies contribute to attaining high-quality healthcare services and the objective of Universal health coverage (1, 2). Students believed that remote services improve patient care and appreciated by patients and clinicians. They were willing to include tele pharmacy services in their future pharmacy practice (3, 4). In the United Kingdom (UK), Digital literacy is becoming increasingly crucial in pharmacy education (5). Evidence on effectively incorporating tele-education into pharmacy curricula is scarce, partly because pharmacy education combines diverse methods: classroom-based learning, simulations, and practical training (6). Students expressed a need for enhanced tele pharmacy, pharmacy management training, and a more substantial alignment between academic content and practical application (3, 7). The epidemic of COVID-19 accelerated the Digital transition in pharmacy education, setting a foundation for sustainable Digital teaching and learning changes that could benefit pharmacy education beyond the crisis (6, 8). A global study revealed that 57% of pharmacy schools lack Digital Health content in their curricula, pointing to a considerable gap in preparing students for Digital Health’s role in modern healthcare (9). Tailored educational tools and supportive learning environments are critical in preparing pharmacy students for successful careers (10–12). The current review aims to gather and evaluate the impact of Digital Health on pharmacy education by examining current practices and identifying gaps to better prepare students for modern healthcare needs.

Pharmacy education and training have evolved with the expanded role of pharmacists to encompass individual patient-centered care and further optimization of therapy and disease management. Digital Health innovations such as tele-pharmacy, telemonitoring, and tele therapeutics places the pharmacy students and faculty to embrace and reflect the recent advancement in pharmacy practice. AI in pharmacy education is transforming how students learn and interact with the material. AI technologies can provide personalized learning experiences, adapt to individual student needs, and enhance the understanding of complex concepts. For example, AI-driven simulations and virtual patients allow students to practice clinical decision-making in a risk-free environment. Additionally, AI can assist in automating administrative tasks, giving educators more time to focus on teaching. This integration of technology not only improves educational outcomes but also prepares future pharmacists to effectively utilize AI in their practice.

### 1.1 What is known?

Pharmacy education now includes Digital tools like EHRs, AR, and tele pharmacy, enhancing student engagement and skills. While students view these tools positively, training in areas like tele pharmacy, mHealth, and AI still needs to be improved.

### 1.2 What is not known?

A standardized Digital Health curriculum needed to foster consistent Digital literacy. Critical gaps include training in AI ethics, data privacy, tele pharmacy, and research on the long-term effects of Digital Health education on clinical performance and patient readiness for tech-driven healthcare warranted.

### 1.3 What will the current study add?

The present scoping review examines the consequences of Digital Health on pharmacy education, identifying shortcomings in the curriculum and the approaches to uniform training of electronic literacy. It explores the assimilation of available technologies like AI, mHealth, and tele pharmacy and finds lacunae between the programs and suitable modes of training. The review emphasizes hands-on training to help students gain competence and confidence. It advocates long-term studies to evaluate real-world impacts and calls for ethics-focused training on AI, promoting better preparation for pharmacy students in Digital healthcare roles.

### 1.4 Rationale

Tele therapeutic, telemedicine, Telehealth, m-health, mobile-health, smart apps, e-health, tele pharmacy, and EHRs are examples of Digital Health technologies that are increasingly integrated into pharmacy education to prepare students for a Digital healthcare environment. While there is evidence of positive outcomes, such as improved clinical decision-making, the research on Digital Health in pharmacy education is scattered and lacks coherence. A scoping review is ideal for exploring this broad area, as it includes diverse study designs and methodologies.

### 1.5 Objective

The current scoping review collates and appraises the impact of Digital Health on pharmacy education, evaluating effects on learning outcomes, skill development, competencies, and readiness for tele pharmacy and Digital Health application transformations. The primary objective was to explore the impact of Digital Health (eHealth, mHealth, e learning, telehealth, AI, telemedicine, tele pharmacy, VR, ML, and AR) in pharmacy education via structured scoping review reporting.

## 2 Methods

The Protocol developed to conduct the current scoping review not registered in PROSPERO. Eligibility criteria the inclusion criteria for this review encompass studies published between 2019 and 2024, focusing on Digital Health interventions in pharmacy education (e-Health, mHealth, e-learning, mobile health, smart apps, telehealth, AI, telemedicine, tele pharmacy, VR, ML, and AR). Any type of study using Digital Health interventions compared to traditional learning methods in pharmacy education with population defined as pharmacy students were deemed eligible for inclusion. Restriction imposed to studies published in English language. The specific outcomes included increased student engagement, active learning, long-term knowledge retention, improved academic performance and high student satisfaction.

The selection period was limited to the past 5 years to capture the most recent developments in Digital pharmacy education. Studies lacking full-text availability or those from non-peer-reviewed sources excluded to ensure the inclusion of high-quality evidence. Studies with multiple outcomes and studies not specific to pharmacy education were excluded from the review.

The information sources for the current review included a comprehensive literature search across multiple databases: PubMed, Cochrane Library, Web of Science, Scopus, CINAHL, and Embase. The search process adhered to the PRISMA, Preferred Reporting Items for Systematic reviews and Meta-Analyses extension for Scoping Reviews (PRISMA-ScR) Checklist (Supplementary Appendix 1). Whereby a systematic approach applied to study identification, screening, and inclusion of relevant articles on Digital Health (smart apps, eHealth, mHealth, e learning, telehealth, AI, ML, telemedicine, tele pharmacy, VR, and AR). A detailed Search strategy applied in EMBASE, PubMed, Scopus, CINHAL, and Cochrane Library. The following keywords and BOOLEEN used (“Digital Health” OR “e-learning” OR “telehealth” OR “eHealth” OR “mHealth” OR “tele-pharmacy” OR “telemedicine” OR “artificial intelligence” OR “virtual reality” OR “augmented reality” OR “big data” OR “machine learning”) AND (“pharmacy education” OR “pharmacists” OR “pharmaceutical care” OR “students”) AND (“interventions” OR “implementation” OR “curriculum” OR “programs” OR “training”). The selection for sources of evidence involved two steps to select articles for the current scoping review. In the initial screening, the titles and abstracts of the included articles used to evaluate their relevance. In the second step, a complete reading of the paper to ensure the eligibility of each study based on the pre-identified inclusion and exclusion criteria. A standardized data charting form developed [using the Joanna Briggs Institute (JBI) data extraction tool] (Supplementary Appendix 2), was tested to ensure its effectiveness in systematically capturing relevant data encompassing smart apps, ML, Digital Health (e-Health, mHealth, e-learning, telehealth, AI, telemedicine, tele pharmacy, VR, and AR). The extracted variables for data items included: study characteristics (author, year, country), intervention details (type of Digital Health tool, duration), outcomes (learning outcomes, student satisfaction), and assessment of the impact on pharmacy education. The critical appraisal of individual sources of evidence (Supplementary Appendix 3) accomplished using the Mixed Methods Appraisal Tool (MMAT) to assess the methodological quality of the selected and included studies. The synthesis of results is performed by thematic analysis, grouping articles based on types of Digital Health interventions and their reported impact on pharmacy education.

## 3 Results

**FIGURE 1:**
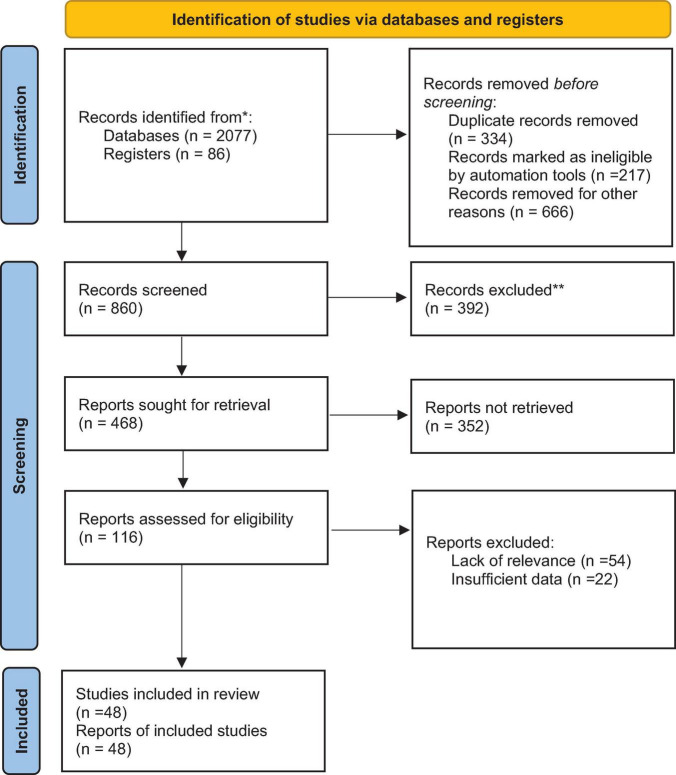
Preferred reporting items for systematic reviews and meta-analyses (PRISMA) 2020 flow diagram for new systematic reviews which included searches of databases and registers only.

For the current review, 2077 identified from databases sources related to the impact of Digital Health in education and 86 from registers. Records removed before screening due to duplicate records (*n* = 334), marked as disqualified (*n* = 217), and (666) for other reasons. Records screened (*n* = 860) while (*n* = 392) records excluded. Reports sought retrieval (*n* = 468), deemed relevant and assessed for eligibility. Reports assessed for eligibility were (*n* = 116) while (*n* = 352) reports not retrieved. Reports excluded for lack of relevance (*n* = 54) and insufficient data (*n* = 22). Ultimately, 47 studies were included in the current scoping review. The others excluded due to lack of relevance or insufficient data or not specific to the impact of Digital Health on pharmacy education. Results of search depicted on the PRISMA flow chart for included studies (Figure 1). The characteristics of sources of evidence of the included articles charted based on crucial information such as the authors and publication year, the study design, the Digital Health interventions, and the outcome measures. A Critical appraisal within sources of evidence of the included articles, adapted for practice settings, the clarity of the Digital Health interventions, and the reliability of outcome assessments related to patient care. Twenty-eight out of forty-seven studies scored highly in participant selection and intervention clarity, while twenty had less clear outcome measures.

### 3.1 Results of individual sources of evidence

The results of individual sources of evidence comprehended that the review synthesizes findings on the integration of Digital Health tools in pharmacy education, organized under five thematic areas. The five thematic areas comprised curriculum integration and transformation in pharmacy education; Digital literacy (competency development); tele pharmacy (remote health services and AI) in education; practical skill development (interactive learning through Digital tools) and, student and faculty perceptions, attitudes, and challenges) in adopting Digital Health. Each theme is examined through observational and original studies.

### 3.2 Theme 1: curriculum integration and transformation in pharmacy education

A study on mHealth applications found that pharmacy students familiar with adherence apps demonstrated more excellent knowledge and acceptance of Digital Health, highlighting the benefits of curriculum alignment with Digital Health advancements (13). Blended learning preferences among pharmacy students in Ireland indicated a strong preference for recorded lectures, but challenges like social isolation highlighted the need for support in technology-enhanced learning (14). Faculty perspectives at United States of America (USA). Pharmacy schools emphasize the positive impact of telehealth integration on student engagement and support for Digital Health tools within the curriculum (15). Studies on curriculum transformation indicate the critical role of Digital Health tools, particularly during the COVID-19 pandemic, in enhancing curriculum flexibility and maintaining educational standards (8). A global survey revealed that only 43% of pharmacy programs offer formal Digital Health training, with those that do report positive impacts on students’ Digital readiness (16). A comparison of EHRs training approaches in USA schools revealed varying comfort levels among students, with extracurricular experience in EHRs further boosting confidence (4). Tele pharmacy readiness was explored at the University of Tennessee Health Science Center, where pharmacy students showed positive attitudes, albeit limited knowledge of tele pharmacy, indicating a need for further training (17). Variations in curriculum quality across Canada and the UK show that students seek more comprehensive Digital Health training to support their professional development (17). Applying the Taba model to rural health courses at the University of Wisconsin-Madison increased student engagement and competence in Digital Health skills, preparing them for rural healthcare (18) (Table 1). The topic of workload and curriculum overload concerns in the use of Digital technology in pharmacy education highlights the challenges faced by both educators and students. With the increasing integration of Digital tools and online resources, there is a risk of overwhelming students with excessive material and assignments. It’s crucial to strike a balance between embracing technology to enhance learning and ensuring that the curriculum remains manageable. Some potential concerns include: (1) Increased expectations: Digital platforms may lead to higher expectations for students to engage with Supplementary material, which could contribute to stress and fatigue. (2) Accessibility issues: Not all students may have equal access to technology, potentially widening the gap between those who can fully benefit from Digital resources and those who cannot. (3) Quality versus quantity: There’s a need to focus on the quality of educational content rather than just adding more resources, ensuring that each component serves a clear educational purpose. (4) Support for educators: Faculty members may require additional training to effectively integrate technology into their teaching strategies without creating an overwhelming workload. Addressing these concerns is essential for optimizing pharmacy education and ensuring that the incorporation of Digital technologies enhances, rather than detracts, the learning experience.

**TABLE 1:** P for population, I for intervention, C for comparison, O for outcomes, and S for study design (PICOs) of selected studies on curriculum integration and transformation in pharmacy education.

References	Study design	Population	Intervention	Comparison	Outcomes
Rehman et al. (12)	Cross-sectional study	Pharmacists and pharmacy students	Evaluation of knowledge and perception of mHealth medication adherence applications	Traditional methods	Assess knowledge levels, perception, and willingness to recommend MApps; identify areas for improvement
Durand et al. (13)	Cross-sectional study	Third- and fourth-year students	Technology-enhanced learning (TEL) during the COVID-19 pandemic	TEL during the COVID-19 pandemic	Student preference for blended learning, with a strong inclination for in-person interactions
Bingham et al. (14)	Exploratory survey	Pharmacy faculty members at a U.S. college of pharmacy	Integration of telehealth education into pharmacy curricula	Standard pharmacy curricula	Faculty perceptions on telehealth, comfort with telehealth topics, and intention to incorporate telehealth
Mirzaian and Franson (7)	Observational case study and process evaluation	Pharmacy students, faculty, and institutions adapting to online education during COVID-19	Implementation of Digital transformation processes	Traditional in-person education and initial responses to pandemic restrictions	Improved Digital capabilities in pharmacy education, sustained educational quality, faculty and student adaptation to online formats
Mantel-Teeuwisse et al. (15)	Cross-sectional study	Pharmacy students, practitioners, and educators worldwide	Implementation and inclusion of Digital Health courses within pharmacy curricula	Those without Digital Health programs	Identification of knowledge gaps, and readiness of pharmacy schools to equip students with Digital Health competencies
Cook et al. (3)	Comparative survey-based study	Pharmacy students at two U.S. institutions	Electronic health record (EHR) training	Varying curricula in EHR training	Students’ perceived readiness and comfort with EHR tasks
Patel et al. (16)	Cross-sectional study	Pharmacy students at the University	Evaluation of pharmacy students’ knowledge, attitudes, and perceptions toward tele pharmacy	Individual perspectives and general attitudes	Insights into students’ knowledge level, perceived effectiveness, and acceptance of tele pharmacy
Park and Min (6)	Mixed-methods study using surveys and semi-structured interviews	Pharmacy students at the University of British Columbia, Canada	Evaluation of Digital Health literacy and technology use in pharmacy education	Comparison based on year of study and exposure to practical experiences	Assessment of self-rate e-health literacy and perceived need for more Digital training
Portillo et al. (17)	Case study	Pharmacy students at the University of Wisconsin-Madison	Implementation of a rural health course using the Taba curriculum model	Case study format	Increased preparedness of students for rural pharmacy practice

EHR, electronic health record; COVID-19, coronavirus disease 2019; e-health literacy, Digital Health literacy; MApps, mHealth medication adherence apps; PICOs, (P) population, (I) intervention, (C) comparison, (O) outcomes, and (S) study design; tele pharmacy, remote delivery of pharmacy services; TEL, technology-enhanced learning.

### 3.3 Theme 2: Digital literacy and competency development

Throughout countries, pharmacy students reported to have varying levels of Digital literacy. The South Indian pharmacy students who used AI for self-medication expressed concerns about the risks associated with self-prescribing, hence emphasizing the need for the provision of guidance on AI literacy in pharmacy education (19). Nottingham University’s co-creation of Digital learning resources with pharmacy and veterinary students enhanced Digital competencies and engagement in Digital Health education (20). Studies in Jordan, Palestine, and the broader Middle East and North Africa (MENA) region identified generally positive attitudes toward AI but noted concerns over data 0 security and ethical implications (21, 22). Malaysian pharmacy students exhibited strong electronic health (eHealth) literacy but expressed doubts about their ability to use Digital Health tools in clinical settings, highlighting the need for curriculum improvements (23). In Turkey, higher eHealth literacy observed among students who use mobile health apps frequently, which underlines the need for repeated engagement with Digital resources regarding Digital competencies (24) (Table 2). Digital literacy initiatives have shown promising results. A study using wearable devices like continuous glucose monitoring (CGM) and blood pressure (BP) monitors demonstrated that students improved significantly in interpreting health data, preparing them for modern Digital Health practices (25). A Digital pharmacy training program using AR significantly enhanced knowledge of diabetes management and practical Digital skills among pharmacy students in Indonesia (26). Digital skills training improved reading comprehension among Indonesian pharmacy students, significantly increasing proficiencies in browsing, presenting, and video conferencing (27). Training with EHRs improved Digital readiness and competency in navigating health records among pharmacy students, with students demonstrating heightened accuracy in clinical tasks, reinforcing the need for Digital training in core pharmacy curricula (28) (Table 2).

**TABLE 2:** P for population, I for intervention, C for comparison, O for outcomes, and S for study design (PICOs) of selected studies on Digital literacy and competency development.

References	Study design	Population	Intervention	Comparison	Outcomes
Nikitha et al. (18)	Cross-sectional observational study	South Indian pharmacy students	Use of artificial intelligence (AI) and internet resources for self-medication	Without reliance on AI and internet	Knowledge, attitudes, and practices regarding self-medication; prevalence of self-medication and associated risks
Chandarana et al. (19)	Reflective survey and evaluation study	Pharmacy and veterinary students at the University of Nottingham	Co-creation of interprofessional Digital learning resources	Evaluation of traditional learning	Increased student engagement, skill development, and curriculum enhancement
Hasan et al. (20)	Cross-sectional study	Pharmacy students and faculty members in Jordan	Knowledge, attitude, and practice (KAP) assessment toward artificial intelligence (AI) in pharmacy	Differences in KAP between students and faculty members	Levels of awareness, attitudes, and factors affecting AI adoption in pharmacy education
Kinny et al. (24)	Exploratory pilot course with pre- and post-surveys	Final-year pharmacy students at Heinrich Heine University	Practical course using wearables for health monitoring	Assessment of outcomes based on pre- and post-surveys	Enhanced Digital Health competencies and knowledge application
Mosleh et al. (21)	Cross-sectional survey	Medical and pharmacy students in Jordan and the West Bank of Palestine	Assessment of knowledge, attitudes, and practices regarding AI programs, including ChatGPT	Comparisons between medical and pharmacy students	Insights into students’ awareness and use of AI in education and differences based on location and specialization
Kurniawan et al. (25)	Quasi-experimental study	Pharmacy students from Indonesia	Use of Android-based augmented reality (AR) models for learning about diabetes mellitus (DM) drug information	Traditional learning methods without AR	Improvement in pharmacy students’ knowledge, engagement, and satisfaction regarding diabetes mellitus drugs
Blebil et al. (22)	Cross-sectional study	Pharmacy students in Malaysia	Assessment of eHealth literacy and use of mobile health (mHealth) applications	Traditional methods	Level of eHealth literacy, usage behavior toward mHealth applications, and confidence in Digital Health skills
Hariyati et al. (26)	Experimental study	Pharmacy students	Digital skills concept integration in learning, specifically focused on reading comprehension	Traditional learning	Improved Digital literacy (proficiencies in LMS, browsing, presenting, video conferencing, and brainstorming
Üstün et al. (23)	Cross-sectional study	Pharmacy students in Istanbul, Turkey	Evaluation of e-health literacy and mobile health application utilization	Comparison across different demographics and usage levels of mobile health apps	Assessment of students’ e-health literacy levels and attitudes toward mobile health app usage
Ives et al. (27)	Experimental study	Doctor of pharmacy students	Use of electronic health record (EHR) technology for processing inpatient medication orders	Prior method using paper-based medication forms	Improved confidence and performance in verifying inpatient medication orders

AI, artificial intelligence; KAP, knowledge, attitude, practice; AR, augmented reality; DM, diabetes mellitus. eHealth, electronic health; EHR, electronic health record; LMS, learning management system; mHealth, mobile health applications.

### 3.4 Theme 3: tele pharmacy, remote health services, and AI in education

The tele pharmacy training in pharmacy programs positively perceived, though challenges remain. Pharmacy students in Saudi Arabia supported tele pharmacy education, though faculty limitations in expertise and resources posed difficulties (29). In the MENA region, students expressed concern over the ethical aspects of AI, primarily related to privacy and potential job displacement (30). A cross-sectional survey of Digital literacy among pharmacy students in Iraq showed that students found ChatGPT beneficial for academic tasks, though it posed concerns over critical thinking skills (31). Indonesian students studying tele pharmacy viewed it positively for improving access but noted that limited prior knowledge was a significant barrier to tele pharmacy adoption (32). A tele pharmacy module developed for pharmacy students in Malaysia enhanced students’ confidence in virtual healthcare, underscoring the value of practical tele pharmacy training (12). Malaysian students showed high awareness and positive attitudes toward tele pharmacy, though they expressed concerns over workload and necessary incentives (33) (Table 3). Original research studies validate the efficacy of tele pharmacy training. A non-inferiority trial in Germany found tele pharmacy training for inhaler techniques as effective as in-person training, with high student satisfaction reported (34). USA pharmacy students view the tele pharmacy as an effective tool to improve healthcare access in rural areas, although relationship building was a noted barrier (35). A study finding revealed that on-campus objective structured clinical examinations (OSCEs) are preferred for interaction and skill assessment. However, virtual OSCEs offer flexibility and could be improved for practical skill training (36). Pharmacy students in Iraq and the United Arab Emirates (UAE) highlighted the value of AI tools like ChatGPT in academic efficiency. However, they were concerned about the influence AI will have on traditional learning skills (37), (Table 3).

**TABLE 3:** P for population, I for intervention, C for comparison, O for outcomes, and S for study design (PICOs) of selected studies on tele pharmacy, remote health services, and AI in education.

References	Study design	Population	Intervention	Comparison	Outcomes
Alsultan et al. (28)	Cross-sectional study	Pharmacy students from five universities in Saudi Arabia	Integration of tele pharmacy education into the pharmacy curriculum	Traditional learning	Students’ knowledge and perceptions of tele pharmacy education
Hasan et al. (29)	Cross-sectional study	Pharmacy students from the Middle East and North Africa (MENA) region	Exploration of attitudes toward AI integration in pharmacy education and practice	Focusing on students’ concerns and influences on attitudes	Insight into ethical concerns and the need for comprehensive AI education in curricula
Imad Mohammed et al. (30)	Qualitative study using face-to-face interviews	Senior pharmacy students at two pharmacy colleges in Iraq	Use of artificial intelligence (AI), particularly ChatGPT, for academic purposes	Traditional study methods	Perceptions of the benefits and drawbacks of AI in enhancing or hindering academic skills
Ali Sherazi et al. (33)	Randomized crossover non-inferiority trial	German pharmacy students in their final semester	Telepharmacy inhaler technique training service	Face-to-face inhaler technique training	Student performance in demonstrating inhaler technique, confidence, and perceptions of tele pharmacy
Alfian et al. (31)	Cross-sectional study	Pharmacy students in Indonesia	Assessment of knowledge, perception, and willingness to provide tele pharmacy services	Internal factors (e.g., smartphone proficiency, age, gender)	Insights into students’ tele pharmacy knowledge, perceptions, and willingness to integrate tele pharmacy in future practice
Isamuddin et al. (11)	Qualitative study	Undergraduate pharmacy students in Malaysia	Development of a tele pharmacy training module	Traditional study methods	Identification of essential components for tele pharmacy training to improve knowledge and skills
Frenzel and Porter (34)	Mixed-method research with post-surveys and thematic analysis	Second- and third-year pharmacy students at two pharmacy schools	Tele pharmacy and telehealth training module	Traditional study methods	Knowledge increases, attitudes, behavioral control, and intent to use telehealth
Kharaba et al. (35)	Randomized controlled head-to-head comparative assessment	Fourth-year pharmacy students at Al Ain University	Comparison between on-campus and virtual OSCE	On-campus OSCE versus virtual OSCE	Student and examiner satisfaction, feasibility, and skill assessment efficacy
Elnaem et al. (32)	Cross-sectional survey	Senior pharmacy students in a Malaysian public university	Assessment of tele pharmacy knowledge, perceptions, and readiness	Internal analysis of responses	Levels of knowledge, perception, and readiness toward tele pharmacy
Arman Rabbani et al. (36)	Descriptive report of a virtual training program	Final-year bachelor of pharmacy students in the UAE	Virtual experiential training across community and hospital pharmacy settings	Traditional in-person experiential training	Successful learning outcomes achieved, though not fully replacing in-person experience

AI, artificial intelligence; MENA, Middle East and North Africa; tele pharmacy, remote delivery of pharmacy services. OSCE, objective structured clinical examination; Tele pharmacy, remote delivery of pharmacy services; tele health, healthcare services delivered remotely.

### 3.5 Theme 4: practical skill development and interactive learning through Digital tools

The use of Digital tools has also contributed to the better development of practical skills. The tele pharmacy practice in Sweden positively supported, with students affirming its usefulness and expecting high technical competence standards in-patient care (3). The introduction of AI teaching tools enjoyed widespread acceptance in many regions, which gives an impression of positive performance when it comes to practical skill training (38). Interactive learning models, including group discussions and problem-solving, fostered better engagement and knowledge retention among pharmacy students in Macedonia (39) (Table 4). Interactive Digital tools have significantly enhanced student engagement in skill-based training. Pharmacy technicians in Spain who trained via Digital electronic learning (e learning) and simulations showed advanced competencies in sterile compounding and non-sterile preparation (40). AR-based diabetes education tools improved understanding and satisfaction among Indonesian students, showing the effectiveness of interactive technologies in pharmacy education (26). Pharmacy students in Germany improved their clinical pharmacy skills, particularly in diabetes management, by using Digital Health tools (41). A Digital pharmacy-training program conducted in Indonesia enhanced students’ practical skills and had high satisfaction rates, indicating the success of Digital tools in practical learning (9). USA pharmacy students in compounding labs using an AR application reported increased engagement and skill retention, demonstrating the value of Digital tools for experiential learning (42). AR and smart glasses in USA pharmacy labs increased student satisfaction and engagement, showing promise in supporting hands-on education (43). My Dispense virtual simulation in the USA effectively engaged students in self-care therapeutics, though no significant changes in confidence were reported (44). Remote experiential learning adaptations in Malaysia using My Dispense simulations helped retain dispensing skills, though face-to-face skills like communication were challenging to replicate (45) (Table 4).

**TABLE 4:** P for population, I for intervention, C for comparison, O for outcomes, and S for study design (PICOs) of selected studies on practical skill development and interactive learning through Digital tools.

References	Study design	Population	Intervention	Comparison	Outcomes
Ros Castellar et al. (39)	Training evaluation and satisfaction survey	Pharmacy technicians in a tertiary hospital	Compounding training program using Digital e-learning and simulation	Traditional study methods	Skill acquisition, satisfaction, and qualification in sterile and non-sterile compounding
Obarcanin et al. (40)	Descriptive study	Final-year pharmacy students	m-Health course focusing on Digital diabetes apps	Traditional learning methods	Improved Digital skills for managing diabetes in clinical pharmacy
Oktianti et al. (8)	Pre-test/post-test training intervention	Pharmacists in Indonesia	Training in Digital media use for Digital pharmacy	Knowledge levels pre- and post-training	Increased knowledge of Digital pharmacy and medication therapy management
Nounou et al. (41)	Cross-over study design conducted across two cohorts	First-year pharmacy students in a pharmaceutical compounding laboratory course	Implementation of a mobile-based augmented reality (AR) application (AmplifiedRx app) in pharmaceutical labs	AR applications are used versus traditional lab methods without AR	Enhanced student engagement, improved understanding and confidence in laboratory skills, and high acceptability of the AR technology
Kapp et al. (42)	Pilot study with pre- and post-surveys	Pharmacy students in a university laboratory course	Augmented reality (AR) environment using smart glasses and mobile devices	Traditional laboratory teaching methods without AR	Student perceptions of AR as a learning aid, user experience, self-efficacy, and anxiety toward technology
Pihl et al. (2)	Qualitative study	Swedish pharmacy students	Education on tele pharmacy and patient communication at a distance	None explicitly mentioned; indirect comparison to traditional face-to-face communication	Students’ preparations for tele pharmacy; their views on the adequacy of current education
Tai et al. (43)	Cohort study	First-year pharmacy students in a self-care therapeutics course	Virtual simulation using MyDispense in self-care therapeutics	Traditional course format without virtual simulation	Frequency of interactions during introductory pharmacy practice experience, student confidence, preceptor-reported student performance
Ameri et al. (37)	Cross-sectional study	Pharmacy students in Iran	Usage of a mobile-based educational application	Traditional learning methods	Behavioral intention to use and long-term acceptance of the LabSafety app
Rahman et al. (44)	Case study	Pharmacy students in Malaysia	Remote experiential learning in community pharmacy	Traditional, on-site community pharmacy experiential learning	Skill acquisition in dispensing, OTC consultations, and overall pharmacy practice
Tomevska Ilievska et al. (38)	Qualitative study with educational assessments	Undergraduate pharmacy students	Interactive teaching models, including group discussions, tutorials, and problem-solving activities	Traditional teaching methods	Increased engagement, active learning, and long-term knowledge retention

AR, augmented reality; mHealth, mobile health applications. Tele pharmacy, remote delivery of pharmacy services; OTC, over the counter; virtual simulation, use of Digital simulations for practical training.

### 3.6 Theme 5: student and faculty perceptions, attitudes, and challenges in adopting Digital Health

Pharmacy students generally have an optimistic but cautious approach toward Digital Health. Students in 12 countries reported positive attitudes toward AI but were apprehensive about its potential impact on job security (46). In the U.K., pharmacy students recognized the importance of Digital literacy education in pharmacy curricula but noted the need for faculty development and curriculum enhancement (5). Pharmacy students in the USA using ChatGPT expressed high satisfaction but desired traditional learning to balance academic tasks (47). Virtual training feedback from Cairo University highlighted high student satisfaction, though session density needed adjustment to improve experiences (48). Digital versus printed poster preferences improved student engagement and accessibility, supporting Digital formats for learning (49). Electronic learning (e learning) well perceived in Mongolia as an alternative to traditional methods, though students preferred a hybrid approach combining Digital and hands-on experiences (50). A telehealth rotation study found that pharmacy students gained valuable experience in inter-professional telehealth collaboration (51). Students at a Virtual Annual Meeting liked the flexibility of Digital learning but preferred hands-on experience for skill building (10) (Table 5). Perception based studies offer solutions to the barriers that can hinder the use of Digital Health. A scoping review pinned 40 important Digital Health topics for medical students taught within knowledge, skills, and attitudes (52) (Table 5).

**TABLE 5:** P for population, I for intervention, C for comparison, O for outcomes, and S for study design (PICOs) of selected studies on student and faculty perceptions, attitudes, and challenges in adopting Digital Health.

References	Study design	Population	Intervention	Comparison	Outcomes
Busch et al. (45)	Cross-sectional study	International pharmacy students	Survey on attitudes toward AI in pharmacy education	Traditional learning methods	Attitudes and preparedness toward AI in pharmacy
Anderson et al. (46)	Cross-sectional study	Pharmacy students in a U.S. PharmD program	Use of ChatGPT for personal, academic, and clinical purposes	Traditional learning methods	Patterns of ChatGPT usage and perceptions regarding its inclusion in curricula
Naguib et al. (47)	Prospective, mixed-methods study	Fifth-year pharmacy students at Egypt	Virtual training on clinical pharmacy using the virtual faculty of pharmacy Cairo University platform	Comparison between pretest and posttest scores pre- and post-virtual training	Improved academic performance and high student satisfaction
Newsom et al. (48)	Cross-sectional study	First- and third-year pharmacy students at Mercer University	Digital poster presentations	Printed poster presentations	Students’ perceptions of learning tools and preferences
Sodnom et al. (49)	Mixed-methods study including surveys and statistical analysis	Undergraduate students	E-learning during the COVID-19 pandemic	Classroom-based traditional learning methods	Evaluation of learning effectiveness, student satisfaction, and challenges of e-learning compared to classroom training
Bautista et al. (50)	Descriptive report on a clinical rotation	Pharmacy and medical students	Interprofessional telehealth rotation for outreach to vulnerable patients	Traditional in-person rotations were not directly compared	Enhanced interprofessional communication, understanding of roles, and telehealth skills
Kelm and Bush (51)	Descriptive report on program development	Pharmacy technician students	Pharmacy technician training program using Digital content	Traditional learning methods	Skills development, certification readiness, and retention
Jenkins et al. (9)	Observational study	Pharmacy students and residency program directors	Examine the effectiveness and perception of PhORCAS	Traditional methods	The critical role of tailored educational tools and supportive learning environments in preparing pharmacy students for successful careers

AI, artificial intelligence; ChatGPT, a language model used for academic and clinical purposes; U. S., United States; e-learning, online learning; PhORCAS, pharmacy online residency centralized application service; tele health, remote healthcare services.

Table 6 depicted the advantages and disadvantages of Digital Health innovations in curriculum integration and transformation in pharmacy education. Further Table 7 have presented examples of AI tools for pharmacy education such as augmented reality (AR), Artificial Intelligence (AI), Natural Language Processing (NLP), Virtual reality (VR). These AI tools can be beneficial for pharmacy education that permit more interactive, personalized, and relevant learning to current practices. More examples of AI brands for Digital Health innovations in pharmacy education were exhibited in Table 8. These brands and platforms can be used in pharmacy education, showing how AI enhances learning with innovative technologies and practical applications.

**TABLE 6:** The advantages and disadvantages of Digital Health innovations in curriculum integration and transformation in pharmacy education.

Advantages of Digital Health innovations in curriculum integration and transformation in pharmacy education:	Disadvantages of Digital Health innovations in curriculum integration and transformation in pharmacy education:
1. Enhanced learning flexibility: Digital tools provide students with the ability to learn at their own pace and access materials at any time, accommodating different learning styles and schedules.	1. Tech-related challenges: not all students may have equal access to technology or a stable internet connection, which could create disparities in learning opportunities.
2. Improved engagement: interactive and multimedia content can make learning more engaging. Gamification and simulation tools can enhance the learning experience by providing practical, real-world scenarios.	2. Learning curve: both educators and students may face a steep learning curve when incorporating new Digital tools into their curriculum, which can lead to frustration and reduced efficacy.
3. Access to up-to-date information: Digital Health innovations allow for the integration of the latest research and current trends in pharmacy practice into the curriculum, ensuring that students are learning the most relevant information.	3. Over-reliance on technology: there is a risk that learners may become overly reliant on Digital tools, potentially diminishing their critical thinking and problem-solving skills.
4. Collaboration opportunities: Digital platforms can facilitate collaboration among students, faculty, and practitioners across different locations, encouraging teamwork and broader knowledge sharing.	4. Quality of content: the availability of vast amounts of online information can lead to concerns about the quality and accuracy of resources used in the curriculum.
5. Skill development: students can develop essential technology skills necessary for modern pharmacy practice, including proficiency in electronic health records, telehealth, and health informatics.	5. Distracted learning environment: the use of Digital devices can lead to distractions, with students being tempted to engage in non-educational activities during lessons.
6. Personalized learning: adaptive learning technologies can tailor educational experiences to meet individual student needs, providing additional resources for those who may be struggling or advanced challenges for those excelling.	6. Assessment challenges: traditional assessment methods may not effectively measure student learning and competency in a Digital environment, necessitating the development of new evaluation strategies. In conclusion, while Digital Health innovations offer numerous benefits for integrating and transforming pharmacy education, careful consideration of the associated challenges is essential to maximize their effectiveness.

**TABLE 7:** Examples of AI tools for pharmacy education.

Examples of artificial intelligence tools for pharmacy education	Summary
1. Simulated patient systems:	AI-powered simulations can create virtual patient scenarios where pharmacy students can practice diagnosis and treatment plans in a safe environment.
2. Personalized learning platforms:	Tools like Edmodo or Blackboard use AI algorithms to adapt course materials and assessments based on individual student performance and learning preferences.
3. AI tutoring systems:	Programs that use AI to provide personalized tutoring in pharmacology and drug interactions, helping students reinforce their learning and address gaps in knowledge.
4. Interactive learning modules:	Virtual reality (VR) and augmented reality (AR) tools that simulate pharmacy practice, allowing students to immerse themselves in real-world environments and scenarios.
5. Drug interaction databases:	AI-enhanced databases help students explore and understand drug interactions and contraindications in real-time.
6. Assessment tools:	AI-based assessment systems that can analyze student responses and provide immediate feedback, helping educators identify areas where students may need additional support.
7. Machine learning for research:	Tools that utilize machine learning to analyze pharmaceutical research data, helping students learn how to conduct data analysis in their projects.
8. Natural language processing (NLP) tools:	These can help students study the latest literature by extracting relevant information and summarizing it efficiently.
9. mHealth	Adherence apps demonstrated more excellent knowledge and acceptance of Digital Health
10. Blended learning and technology-enhanced learning	Recorded lectures
11. Telehealth integration	Student engagement

AR, augmented reality; AI, artificial intelligence: NLP, natural language processing: VR, virtual reality. The above are some examples of AI tools that can be beneficial for pharmacy education. These tools make education more interactive, personalized, and relevant to current practices.

**TABLE 8:** Examples of AI brands for and Digital Health innovations for pharmacy education.

Examples of artificial intelligence brands for pharmacy education	Summary information on the type of AI brands and Digital Health innovations
1. BM Watson health	Utilizes AI to analyze health data and can be used in pharmacy education for insights into drug interactions and patient care.
2. BioSymetrics	Offers AI-driven insights for drug discovery that can be integrated into educational programs for pharmacy students.
3. GNS healthcare	Provides machine learning technology to simulate disease and treatment, useful for pharmacy education focusing on personalized medicine.
4. Aidoc	Although primarily focused on radiology, their AI tools can help educate pharmacy students on imaging and medication management in diagnostic scenarios
5. GRAIL	While focused on early cancer detection, their technology can serve as a case study in pharmacy education about the role of pharmacists in oncology.
6. CureMetrix	Uses AI to support breast cancer screening, providing pharmacy students insight into the importance of medication management in oncology.
7. Proteus Digital Health	Known for their Digital pills, they incorporate AI to improve medication adherence and can serve as a case study in pharmacy education.

The above are examples of AI brands and platforms used in pharmacy education, showing how AI enhances learning with innovative technologies and practical applications.

## 4 Discussions

### 4.1 Discussions and synthesis of results

In synthesizing these results, it became evident that Digital Health technologies are transforming pharmacy education. Digital Health enhances pharmacy education by improving skills, engagement, and confidence with tools like AI, AR, and EHRs. However, better curriculum integration, Digital literacy training, and ethical guidance are needed. Tele pharmacy and AI improve access but raise concerns about workload and critical thinking. Students value a mix of Digital and hands-on learning, calling for updated curricula and more robust faculty support. Some hands-on skills remain challenging to teach remotely.

#### 4.1.1 Curriculum integration and transformation in pharmacy education (curriculum development)

The integration of Digital Health into pharmacy education curricula shows significant advancements, driven by responses to COVID-19 and the increasing demands of modern healthcare. Findings from studies across the USA, Malaysia, and Ireland illustrate a shared commitment to rapidly incorporating Digital Health tools like EHRs and tele pharmacy into pharmacy programs, albeit through differing approaches: the USA focus on meeting competency standards. At the same time, Malaysia and Ireland emphasize blended and fully remote models to address regional needs (8, 14, 45). Studies also reveal common challenges in maintaining interpersonal skill development in fully remote setups, highlighting the importance of hybrid learning approaches for skill retention (18).

#### 4.1.2 Digital literacy and competency development (knowledge and competencies)

Digital literacy differs widely across pharmacy curricula, with research from Turkey, Malaysia, and MENA finding intermediate eHealth literacy levels among students, underscoring the importance of structured training (21, 23, 24). Notably, USA, and Indonesian studies demonstrate the successful use of AR and wearables to engage students in practical applications, bridging theory and real-world skills (25, 26). Hands-on EHR training also improves comprehension and data interpretation skills, showing that immersive Digital tools can effectively build essential pharmacy competencies (28). That aligns with previous reviews that examine Digital Health education, highlighting similar gaps in standardized curricula and calling for consistent competency frameworks (53, 54).

#### 4.1.3 Tele pharmacy, remote health services, and AI in education (skills development)

Tele pharmacy and AI training are emerging areas, yet programs vary significantly in their adoption and focus. In Germany and the USA, tele pharmacy incorporated to prepare students for remote services, while Saudi Arabia and Indonesia show early interest with limited integration (29, 32, 34, 35). MENA region students express AI-related ethical concerns, contrasting with Western students who focus more on practical applications (30, 46). The findings underscore a gap, where Western programs lead Digital integration, while Middle Eastern and Asian programs prioritize foundational tele pharmacy education to address local needs (12, 36). The impact of Digital Health innovations on pharmacy education can be framed through the lens of several key concerns regarding artificial intelligence (AI), which can resonate as a unified theme across different regions.

The impact of Digital Health Innovations on pharmacy education concerns about AI (privacy, self-medication, overreliance) may provide some insights as unified strategy across regions. Firstly, the concern of privacy, as AI systems integrate into pharmacy practice, the handling of sensitive patient data becomes crucial. Pharmacy education must address the ethical implications and legal responsibilities surrounding patient privacy. Students should be taught the importance of data protection and the implications of breaches, emphasizing the need for robust systems to safeguard personal health information, regardless of regional regulations. Secondly, with the rise of Digital Health tools, patients increasingly engage in self-medication based on online information. Pharmacy education should highlight the role of pharmacists in guiding patients to make informed decisions about their health. A unified educational approach can cultivate a global understanding of the risks associated with self-medication and the pharmacist’s role in patient education, promoting safer practices internationally. Lastly, the convenience of AI and Digital Health tools may lead to overreliance on these technologies, potentially undermining clinical judgment and traditional pharmaceutical knowledge. Pharmacy curricula should balance Digital Health innovations with core competencies, ensuring that future pharmacists retain critical thinking skills and the ability to make informed decisions without solely depending on technology.

By addressing these concerns through a unified theme of education, pharmacy programs can prepare students to navigate the complexities of Digital Health innovations responsibly. This holistic approach fosters a global community of pharmacists equipped to adapt to technological advancements while prioritizing patient welfare and ethical practices.

#### 4.1.4 Practical skill development and interactive learning through Digital tools (skills and competencies development)

Interactive Digital tools are increasingly integral to skill development in pharmacy education. In Germany and the USA, mHealth and AR used to enhance engagement, and similar successes reported with AR in Indonesia and Spain, particularly in compounding labs (26, 40, 41, 43). Malaysian remote experiential training highlights challenges in replicating interpersonal interactions, whereas AR-based training preserves hands-on learning benefits (45). These findings collectively support hybrid-learning models to achieve optimal skill acquisition.

#### 4.1.5 Student and faculty perceptions, attitudes, and challenges in adopting Digital Health (student and faculty development)

Student and faculty perceptions reveal both enthusiasm and caution toward Digital Health technologies. Positive feedback includes increased engagement and skill development, especially with tools like ChatGPT and EHRs in USA, and Iraqi programs (28, 31). However, ethical and workload concerns regarding AI and tele pharmacy training in MENA and Southeast Asia highlight differing cultural perspectives on Digital Health adoption (21, 33). A previous review underscores ethical challenges in incorporating AI into pharmacy education, including tool generalizability and legal implications (54). Studies investigating a Digital and traditional learning approach in countries like Mongolia suggest that while Digital platforms offer theoretical learning comparable to a classroom, skills acquisition remains best in practical settings (50). These findings suggest that one potential way to increase the effectiveness of Digital Health training across several educational contexts is by using Digital and analogue tools simultaneously.

The benefits of Artificial Intelligence in pharmacy education are numerous and can significantly enhance the learning experience for students and improve the overall educational process. Via personalized learning AI can tailor educational content to meet individual students’ needs, helping them grasp complex concepts at their own pace. This customization can lead to improved understanding and retention of syllabi. Further, interactive learning environments provided by AI-powered tools can provide immersive simulations and interactive scenarios that make learning more engaging. This hands-on approach can help students apply theoretical knowledge in practical situations. AI can assist students in quickly finding relevant research papers, case studies, and clinical guidelines, enhancing their ability to stay updated with the latest developments in pharmacy (access to vast resources). AI can streamline the assessment process by providing instant feedback on quizzes and assignments (assessment and feedback). This timely information allows students to identify areas where they need improvement and foster continuous learning. AI can analyze trends and patterns in student performance, helping educators identify at-risk students early and implement targeted interventions to support their success (predictive analytics). AI can aid in drug discovery and pharmacological research, providing students with valuable insights into the latest technologies and methodologies being used in the field. AI can assist with administrative responsibilities, allowing educators to spend more time focusing on teaching and mentoring students. AI platforms can facilitate collaboration among students (collaborative learning), enabling them to work on group projects and share knowledge more effectively, regardless of their geographical locations. By integrating AI into pharmacy education, institutions can enhance educational experience, better prepare students for their careers, and ultimately improve healthcare outcomes (18, 20, 21, 29, 30, 33).

The benefits of telepharmacy in pharmacy education are numerous and impactful. First, tele pharmacy can enhance access to educational resources and expertise, allowing students in remote or underserved areas to connect with experienced pharmacists and educators. This can lead to a more comprehensive and varied learning experience. Additionally, tele pharmacy facilitates the use of technology and Digital tools, making pharmacy education more interactive and engaging. Students can participate in virtual simulations, webinars, and online discussions, which can help them develop critical thinking and problem-solving skills in real-time scenarios. Moreover, tele pharmacy can also promote flexibility in learning. Students can schedule their classes or meetings around their availability, making it easier for them to balance their studies with other commitments. Furthermore, this approach encourages collaboration among peers and professionals across various locations. Students can engage in group projects and discussions with individuals from diverse backgrounds, enriching their educational experience and preparing them for teamwork in their future careers. Lastly, tele pharmacy can improve patient care training by allowing students to observe and participate in consultations and medication management scenarios remotely. This exposure is invaluable as it reflects the changing landscape of pharmacy practice and prepares students for the realities of modern healthcare. Overall, the integration of tele pharmacy into pharmacy education is a promising development that can enhance learning, accessibility, and the overall quality of education for future pharmacists (11, 28, 31, 34).

In summary the current scoping review outlined the importance of Digital Health on pharmacy education, influence on learning outcomes, pertinent skill development, and deemed |competencies, for the transformations.

### 4.2 Implementing Digital Health innovations in pharmacy education

Implementing Digital Health innovations in pharmacy education presents several challenges that can be thought of for structured implementation process: (1) Curriculum Integration: One of the primary challenges is incorporating Digital Health technologies into the existing pharmacy curriculum. There needs to be an alignment between traditional pharmacy education and emerging Digital Health trends, which often require curriculum redesign and workload distribution. (2) Technological Infrastructure: Many educational institutions may lack the necessary technological infrastructure to support Digital Health innovations. This includes both hardware (e.g., computers, software) and reliable internet access. (3) Faculty Development and Training: Educators may not have the requisite knowledge or skills to teach Digital Health innovations effectively. Providing adequate training and resources for faculty members is crucial to ensure they can guide students in this area. (4) Funding and Resources: Implementing new Digital Health technologies often requires significant investment. Budget constraints can limit the ability of institutions to adopt and sustain these innovations. (5) Student Engagement: Engaging students with Digital Health tools can be challenging. It’s essential to ensure that students see the value of these innovations in their future practice to foster interest and participation. (6) Regulatory and Compliance Issues: Navigating the regulatory landscape associated with Digital Health can be complex. Pharmacy education programs need to stay updated with changes in laws, AI ethics, and regulations governing Digital Health practices. (7) Interprofessional Collaboration: Digital Health often requires collaboration across various healthcare disciplines. Facilitating interprofessional education to prepare pharmacy students for a collaborative healthcare environment can be difficult but is essential. (8) Assessment of Learning Outcomes: Developing appropriate metrics to assess the effectiveness of Digital Health training in pharmacy education can be challenging. Institutions need to ensure that learning outcomes are met and that students are adequately prepared for real-world applications and regional disparities. Addressing these challenges requires a concerted effort from educational institutions, healthcare organizations, and stakeholders in the pharmacy field to create an environment conducive to the successful implementation of Digital Health innovations.

### 4.3 Summary of evidence

**FIGURE 2:**
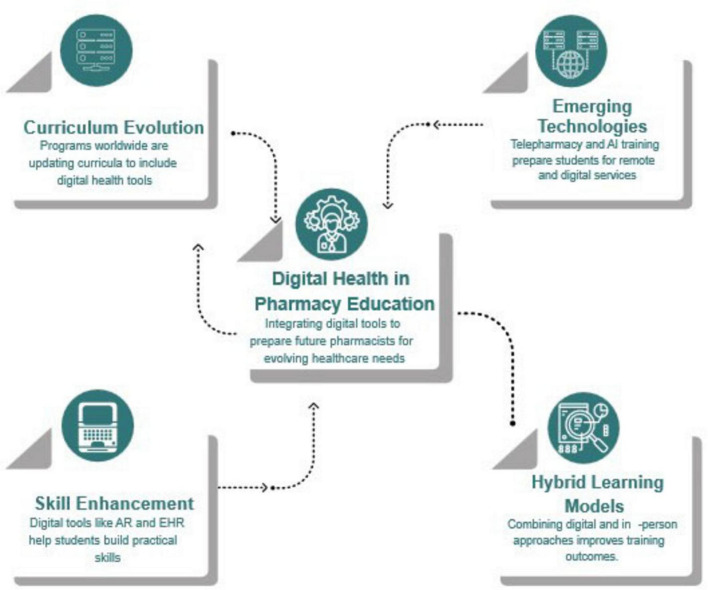
Summary of impact of Digital Health on pharmacy education.

Students obtain excellent proficiency in tele pharmacy and Digital literacy due to the introduction of health technologies in pharmacy training. It is, above all, encouraging, but potential issues such as regional disparities, AI ethics, workload efficiency, energy and level of organization of students and teachers deserve further emphasis. The main key findings illustrated in (Figure 2). The structured results of the impact of Digital Health innovation on pharmacy education have led to significant advancements in the field. These innovations have not only enhanced the quality of education but have also instilled a strong sense of confidence in the findings derived from this research. By systematically analyzing the effects of Digital tools and technologies, educators and practitioners have been able to identify effective teaching methods, improve engagement, and ultimately prepare pharmacy students for the evolving landscape of healthcare. This quality work serves as a solid foundation for future studies and applications, ensuring that the integration of Digital Health in pharmacy education continues to yield positive outcomes (Tables 1–5).

The value added by the current scoping review versus prior reviews, contributes significantly to the existing literature on Digital Health innovation in pharmacy education by providing a comprehensive synthesis of recent advancements and trends. Unlike prior reviews, which may have focused on specific aspects or limited timeframes, this review encapsulates a broader range of studies and evidence, highlighting the evolving nature of Digital tools and their implications for educational practices. Additionally, this review addresses gaps identified in earlier works, such as the practical application of Digital Health innovations in curriculum development and student learning outcomes. By analyzing a wider variety of Digital Health technologies and their educational impacts, the current review offers a more nuanced understanding of how these innovations can enhance pharmacy education. Furthermore, it emphasizes the importance of interdisciplinary collaboration and the integration of Digital Health into pharmacy training, providing actionable insights for educators and policymakers. Overall, this scoping review not only updates the discourse but also sets the stage for future research in the field.

### 4.4 The significance for the impact of versatile Digital Health innovations on pharmacy education

The significance of versatile Digital Health innovations on pharmacy education is profound and multifaceted. As the healthcare landscape continues to evolve, pharmacy education must adapt to incorporate new technologies that enhance learning and improve patient care. The current review summarizes major significance in this context: (1) Enhanced Learning Opportunities: Digital Health innovations provide access to a wealth of resources and learning tools. This includes online courses, virtual simulations, and interactive platforms that engage students in real-world scenarios, making education more dynamic and accessible. (2) Inter-professional Collaboration: Digital Health tools often facilitate communication and collaboration among different healthcare professionals. Pharmacy education that incorporates these technologies prepares students to work effectively in interdisciplinary teams, essential for coordinated patient care. (3) Data-Driven Decision Making: With the rise of big data in healthcare, pharmacy education is increasingly integrating data analysis into curricula. Understanding how to interpret and leverage data is crucial for pharmacists to make informed clinical decisions and improve patient outcomes. (4) Remote Patient Monitoring: Innovations in telehealth and remote monitoring allow pharmacy students to understand modern patient management techniques. Learning to utilize these technologies prepares students to manage medication therapy and patient consultations effectively, regardless of location. (5) Focus on Personalized Medicine: Digital Health innovations are at the forefront of personalized medicine, which tailors’ treatment to individual patients. Pharmacy education that emphasizes these innovations helps students understand the importance of customizing medication regimens based on genetic, environmental, and lifestyle factors. (6) Adaptability to Change: The fast pace of technological advancements requires pharmacy students to be adaptable and agile learners. Incorporating these innovations into education fosters a mindset that embraces continual learning and adaptability in practice. Overall, the integration of versatile Digital Health innovations into pharmacy education not only enhances the learning experience but also ensures that future pharmacists are well equipped to thrive in a rapidly changing healthcare environment.

### 4.5 Limitations

Limitations included potential biases in the selection of studies and the included studies published only between 2019 and 2025, which may have missed other studies that would have affected the result. Further the challenge of measuring the long-term impact of Digital Health training on student outcomes and professional practice may limit the findings. As a scoping review, the assessment of methodological quality was limited. Generally, during conducting the current scoping review on the impact of versatile Digital Health innovations on pharmacy education, several limitations encountered. (1) Variability in Digital Health Innovations: The wide range of Digital Health tools and technologies can make it difficult to assess their specific impacts consistently. Variations in features, target populations, and usage can lead to inconsistent findings. (2) Limited Evidence Base: There may be a lack of empirical studies specifically focusing on the impact of Digital Health innovations on pharmacy education. Many tools have been evaluated in clinical settings rather than educational contexts. (3) Quality of Included Studies: The quality and rigor of the studies that are included in the review can vary significantly. Some may not adhere to strong methodological standards, affecting the overall reliability of findings. (4) Publication Bias: Studies with positive findings more likely published. This can skew the review results since it may not represent negative outcomes or the lack of impact. (5) Rapidly Evolving Field: Due to the fast-paced nature of technological advancements, findings can quickly become outdated. This poses challenges in ensuring that the review is current and relevant. (6) Diversity of Educational Contexts: Pharmacy education varies significantly across different institutions and regions, which can affect how Digital Health innovations are integrated and their subsequent impacts. (7) Stakeholder Perspectives: The review might not adequately capture the perspectives of all stakeholders involved in pharmacy education, such as educators, students, and practitioners, which can limit understanding of the full impact. (8) Integration Challenges: Identifying how Digital Health innovations integrated into existing curricula and the challenges faced during this process can be difficult to assess. (9) Longitudinal Effects: Understanding the long-term impact of Digital Health innovations may require longitudinal studies, which are often underrepresented in the literature. Addressing these limitations in future reviews is crucial for drawing meaningful conclusions about the role of Digital Health innovations in enhancing pharmacy education.

## 5 Conclusion

The current scoping review emphasized that the integration of Digital Health in pharmacy education enhances the students’ readiness to form future professional pharmacy careers. Further it emphasized the importance of faculty developing, workload integration, regional disparities, and ethical concerns with AI and challenges in hybrid learning. Digital Health has been an innovator in pharmacy students’ education, equipping them with the skills and competences required in today’s healthcare environment. However, faculty development, curriculum gaps, workload integration, insufficient telehealth training, regional disparities, and inadequate AI ethics instruction all point to the need for adequate and relevant curriculum modifications to qualify graduates to deal with Digital healthcare challenges. Critical gaps remain in standardized curricula for Digital Health in pharmacy profession, incorporating comprehensive telehealth training, and increasing the emphasis on emerging technologies such as AI, ML, Large language Models (LLMs) and versatile mHealth tools. Addressing these gaps will ensure pharmacy graduates are well equipped to navigate and lead in a digitally driven healthcare environment.

## References

[1] Digital Health. Available online at: https://www.who.int/health-topics/digital-health/#tab=tab_1recommendations on digital interventions for health system strengthening (accessed October 29, 2024). 2024

[2] Pihl R Eriksson I Sporrong S Wettermark B. *Are Swedish Pharmacy Students Prepared for Patient Communication at a Distance?: A Focus Group Study on Pharmacy Students’ Views and Opinions on Telepharmacy and Pharmacy Education, with a focus on communication at a distance.* (2021). Available online at: https://urn.kb.se/resolve?urn=urn:nbn:se:uu:diva-446762 (accessed October 24, 2024).

[3] Cook K Cochran G Gali H Hatch T Awdishu L Lander L. Pharmacy students’ readiness to use the electronic health record: a tale of two institutions. *Curr Pharm Teach Learn.* (2021) 13:327–32. 10.1016/j.cptl.2020.11.005 33715792

[4] Alowais M Nazar H Tolley C. Digital literacy education for UK undergraduate pharmacy students: a mixed-methods study. *Int J Pharm Pract.* (2024) 32:413–9. 10.1093/ijpp/riae040 39180520

[5] Frenzel J Porter A. The need to educate pharmacy students in telepharmacy and telehealth. *Am J Pharm Educ.* (2021) 85:8566. 10.5688/ajpe8566 34615629 PMC8500290

[6] Park J Min J. Exploring Canadian pharmacy students’ e-health literacy: a mixed method study. *Pharm Pract.* (2020) 18:1747. 10.18549/PharmPract.2020.1.1747 32256899 PMC7104795

[7] Mirzaian E Franson K. Leading a digital transformation in pharmacy education with a pandemic as the accelerant. *Pharmacy.* (2024) 9:19. 10.3390/pharmacy9010019 33445718 PMC7839048

[8] Oktianti D Bangsa J Hati A. Training on the use of digital media for implementation digital pharmacy for pharmacist at Salatiga in supporting the industrial revolution 4.0. *Community Empowerment.* (2022) 7:2091–9. 10.31603/ce.7939

[9] Jenkins Z Cole J Chen A Harper N Krauss Z. The role of PhORCAS database functions vs. traditional curriculum vitae in the pharmacy residency application cycle. *Am J Pharm Educ.* (2020) 84:819. 10.5688/ajpe8220 32665732 PMC7334344

[10] Chen J Yang J. Development and application of workbook teaching materials – taking the curriculum construction of pharmacy and traditional chinese medicine pharmacy in higher vocational colleges of pharmaceutical specialty group as an example. *Educ Reform Dev.* (2021) 3:5–9. 10.26689/erd.v3i1.2612

[11] Isamuddin N Elnaem M Kamil T Nazar N. Identification of teaching and learning components of a telepharmacy training module for undergraduate pharmacy students in Malaysia: a qualitative study. *Pharm Educ.* (2023) 23:505–13. 10.46542/pe.2023.231505513

[12] Rehman W Thanganadar H Idrees S Mehmood A Azeez F Almaimani H Knowledge and perception of mHealth medication adherence applications among pharmacists and pharmacy students in Jazan, Kingdom of Saudi Arabia. *PLoS One.* (2024) 19:e0308187. 10.1371/journal.pone.0308187 39213299 PMC11364248

[13] Durand E Kerr A Kavanagh O Crowley E Buchanan B Bermingham M. Pharmacy students’ experience of technology-enhanced learning during the COVID-19 pandemic. *Explor Res Clin Soc Pharm.* (2023) 9:100206. 10.1016/j.rcsop.2022.100206 36471895 PMC9714125

[14] Bingham J Axon D. Telehealth integration into pharmacy practice curricula: an exploratory survey of faculty perception. *Pharmacy.* (2023) 11:110. 10.3390/pharmacy11040110 37489341 PMC10366875

[15] Mantel-Teeuwisse A Meilianti S Khatri B Yi W Azzopardi L Gómez J Digital health in pharmacy education: preparedness and responsiveness of pharmacy programmes. *Educ Sci.* (2021) 11:296. 10.3390/educsci11060296

[16] Patel K. Assessment of knowledge, attitude, perception of pharmacy students towards telepharmacy. *Appl Res Projects.* (2021) 75:72. 10.21007/chp.hiim.0072

[17] Portillo E Look K Mott D Breslow R Kieser M Gallimore C. Intentional application of the taba curriculum model to develop a rural pharmacy practice course. *Innov Pharm.* (2020) 11:2089. 10.24926/iip.v11i1.2089 34017632 PMC8132528

[18] Nikitha B Roopa K Kynshi S Chauhan R Girish B Srinivasan R. Artificial intelligence and internet influence on drug utilization: exploring self-medication trends in South Indian pharmacy students. *Intelligent Pharm.* (2024) 2:814–20. 10.1016/j.ipha.2024.06.001

[19] Chandarana P Rickaby R Sonnex K Allegrucci C Garcia-Ara A. Student co-creation of digital learning resources: an evaluation and reflection of veterinary pharmacy and care home pharmacy interprofessional education packages. *Student Engag High Educ J.* (2024) 5:203–27.

[20] Hasan H Jaber D Al Tabbah S Lawand N Habib H Farahat N. Knowledge, attitude and practice among pharmacy students and faculty members towards artificial intelligence in pharmacy practice: a multinational cross-sectional study. *PLoS One.* (2024) 19:e0296884. 10.1371/journal.pone.0296884 38427639 PMC10906880

[21] Mosleh R Jarrar Q Jarrar Y Tazkarji M Hawash M. Medicine and pharmacy students’ knowledge, attitudes, and practice regarding artificial intelligence programs: jordan and west bank of palestine. *Adv Med Educ Pract.* (2023) 14:1391–400. 10.2147/AMEP.S433255 38106923 PMC10721701

[22] Blebil A Dujaili J Mohammed A Loh L Chung W Selvam T Exploring the eHealth literacy and mobile health application utilisation amongst Malaysian pharmacy students. *J Telemed Telecare.* (2024) 29:58–71. 10.1177/1357633X221077869 35188826

[23] Üstün G Söylemez S Uçar N Sancar M Okuyan B. Assessment of the pharmacy students’ e-health literacy and mobile health application utilization. *J. Res. Pharm.* (2022) 24:23–9. 10.35333/jrp.2020.125

[24] Kinny F Schlottau S Ali Sherazi B Obarcanin E Läer S. Digital health in pharmacy education: elective practical course integrating wearable devices and their generated health data. *Explor Res Clin Soc Pharm.* (2024) 15:100465. 10.1016/j.rcsop.2024.100465 38983639 PMC11231589

[25] Kurniawan A Puspita N Yusmaniar T Rajendra F. The effectiveness of using digital applications for diabetes mellitus with augmented reality models as learning media in pharmacy education. *Pharm Educ.* (2023) 23:53–9. 10.46542/pe.2023.232.5359

[26] Hariyati N. Impact of the digital skills concept in society 5.0 on pharmacy students reading comprehension. *Int Conf Government Educ Manag Tourism.* (2022) 1.

[27] Ives A Tucker S Trovato J. Using electronic health record technology to teach inpatient medication order verification to pharmacy students. *Am J Pharm Educ.* (2020) 84:aje7534. 10.5688/ajpe7534 32934381 PMC7473233

[28] Alsultan M Baraka M Alahmari A Elrggal M Mahmoud M Alrasheed M Knowledge and perception of pharmacy students toward telepharmacy education in Saudi Arabia. *Healthcare.* (2024) 12:1806. 10.3390/healthcare12181806 39337147 PMC11431270

[29] Hasan H Jaber D Khabour O Alzoubi K. Perspectives of pharmacy students on ethical issues related to artificial intelligence: a comprehensive survey study. *Res Sq.* (2024) rs.3.rs-4302115. 10.21203/rs.3.rs-4302115/v1 38746156 PMC11092854

[30] Imad Mohammed S Lateef Jasim A Azeez Al-Jumaili A Mudher Mikhael E Zuhair Ali F. Perceptions of senior pharmacy students towards the impact of artificial intelligence on university education and scientific writing: a qualitative study. *Al-Rafidain. J Med Sci.* (2024) 6:142–6. 10.54133/ajms.v6i1.538

[31] Alfian S Khoiry Q Andhika A Pratama M Pradipta IS Kristina SA Knowledge, perception, and willingness to provide telepharmacy services among pharmacy students: a multicenter cross-sectional study in Indonesia. *BMC Med Educ.* (2024) 23:800. 10.1186/s12909-023-04790-4 37884985 PMC10601297

[32] Elnaem M Akkawi M Al-Shami A Elkalmi R. Telepharmacy knowledge, perceptions, and readiness among future malaysian pharmacists amid the COVID-19 pandemic. *Indian J Pharm Educ Res.* (2022) 56:9–16. 10.5530/ijper.56.1.2

[33] Ali Sherazi B Sayyed S Möllenhoff K Läer S. Telepharmacy versus face-to-face approach in providing inhaler technique training service: a non-inferiority assessment among German pharmacy students. *Integr Pharm Res Pract.* (2024) 13:165–80. 10.2147/IPRP.S468881 39318441 PMC11421451

[34] Frenzel J Porter A. Design and assessment of telepharmacy and telehealth training in two pharmacy programs. *Am J Pharm Educ.* (2023) 87:aje8800. 10.5688/ajpe8800 35260413 PMC10159514

[35] Kharaba Z AlAhmad M Ahmed Elnour A Abou Hajal A Abumweis S Ghattas M. Are we ready yet for digital transformation? Virtual versus on-campus OSCE as assessment tools in pharmacy education. a randomized controlled head-to-head comparative assessment. *Saudi Pharm J.* (2023) 31:359–69. 10.1016/j.jsps.2023.01.004 36718383 PMC9876029

[36] Arman Rabbani S Wadhwa T Sridhar S Shareef J Anwer Ali A Rao P. Experiential training during COVID-19 pandemic: a virtual attachment experience from a college of pharmaceutical sciences in the United Arab Emirates Background and context. *Pharm Educ.* (2021) 21:51–5. 10.46542/pe.2021.211.5155

[37] Ameri A Khajouei R Ameri A Jahani Y. Acceptance of a mobile-based educational application (LabSafety) by pharmacy students: an application of the UTAUT2 model. *Educ Inf Technol.* (2020) 25:419–35. 10.1007/s10639-019-09965-5

[38] Tomevska Ilievska E Tonic Ribarska J Trajkovic Jolevska S Ancevska Netkovska K Tofovikj Kjamilova M. *Interactive Models in University Teaching: Application in Pharmacy Education.* (2019). Available from: https://repository.ukim.mk:443/handle/20.500.12188/9903 (accessed October 24, 2024).

[39] Ros Castellar F Ruano Encinar M Pérez Robles T García Vázquez N Casado Abad G Sánchez Rubio L Training program in compounding for pharmacy technicians through a digital platform and simulation techniques. *Farm Hosp.* (2024): 10.1016/j.farma.2023.07.019 Online ahead of print. 38480046

[40] Obarcanin E Ali-Sherazi B Dabidian A Schlottau S Deters M Läer S. Introducing m-health and digital diabetes apps in clinical pharmacy education in Germany. *J Diabetes Clin Res.* (2022) 4:17–9. 10.33696/diabetes.4.051

[41] Nounou M Eassa H Orzechowski K Eassa H Edouard J Stepak N Mobile-based augmented reality application in pharmacy schools implemented in pharmaceutical compounding laboratories: students’ benefits and reception. *Pharmacy.* (2024) 10:72. 10.3390/pharmacy10040072 35893710 PMC9326741

[42] Kapp K Sivén M Laurén P Virtanen S Katajavuori N Södervik I. Design and usability testing of an augmented reality (AR) environment in pharmacy education—presenting a pilot study on comparison between AR smart glasses and a mobile device in a laboratory course. *Educ Sci.* (2022) 12:854. 10.3390/educsci12120854

[43] Tai M Rida N Klein K Diez H Wells T Kippes K Impact of virtual simulation in self-care therapeutics course on introductory pharmacy practice experience self-care encounters. *Curr Pharm Teach Learn.* (2020) 12:74–83. 10.1016/j.cptl.2019.10.015 31843168

[44] Rahman N Nazar N Elnaem M. Experiential learning in community pharmacy: online and remote teaching experience in Malaysian higher education. *Pharm Educ.* (2020) 20:29–30. 10.46542/pe.2020.202.2930

[45] Busch F Hoffmann L Truhn D Palaian S Alomar M Shpati K International pharmacy students’ perceptions towards artificial intelligence in medicine-A multinational, multicentre cross-sectional study. *Br J Clin Pharmacol.* (2024) 90:649–61. 10.1111/bcp.15911 37728146

[46] Anderson H Kwon S Linnebur L Valdez C Linnebur S. Pharmacy student use of ChatGPT: a survey of students at a U.S. school of pharmacy. *Curr Pharm Teach Learn.* (2024) 16:102156. 10.1016/j.cptl.2024.102156 39029382

[47] Naguib S AlSetohy W Sabry N. Virtual clinical pharmacy training in the era of COVID-19: a report on undergraduate students’ perceptions and academic performance. *Curr Pharm Teach Learn.* (2023) 15:8–18. 10.1016/j.cptl.2023.02.002 36898889 PMC9968616

[48] Newsom L Miller S Chesson M. Use of digital vs printed posters for teaching and learning in pharmacy education. *Am J Pharm Educ.* (2021) 85:8307. 10.5688/ajpe8307 34315702 PMC8341229

[49] Sodnom B Tudevdagva U Luvsandorj T Erdenechimeg S. Comparison of E-learning and classroom training for bachelor students of traditional medicine. *Article Int J Integr Technol Educ.* (2021) 10:55–64. 10.5121/ijite.2021.10205

[50] Bautista C Huang I Stebbins M Floren L Wamsley M Youmans S Development of an interprofessional rotation for pharmacy and medical students to perform telehealth outreach to vulnerable patients in the COVID-19 pandemic. *J Interprof Care.* (2020) 34:694–7. 10.1080/13561820.2020.1807920 32917114

[51] Kelm M Bush P. Digital content delivery in a pharmacy technician training program in a health system. *Am J Health Syst Pharm.* (2024) 77:295–9. 10.1093/ajhp/zxz255 31696923

[52] Khurana M Raaschou-Pedersen D Kurtzhals J Bardram J Ostrowski S Bundgaard J. Digital health competencies in medical school education: a scoping review and Delphi method study. *BMC Med Educ.* (2022) 22:129. 10.1186/s12909-022-03163-7 35216611 PMC8881190

[53] Permata Sari K Maria N Arrum Kusumawardani L Wilda Risni H Farhanah Syafhan N Nur Fauziyyah A. Education and training for improving pharmacist’s telepharmacy competencies: a scoping review (Pendidikan dan pelatihan untuk peningkatan kompetensi apoteker terkait telefarmasi: scoping review). *J Ilmu Kefarmasian Indonesia.* (2023) 21: 293–9.

[54] Abdel Aziz M Rowe C Southwood R Nogid A Berman S Gustafson K. A scoping review of artificial intelligence within pharmacy education. *Am J Pharm Educ.* (2024) 88:100615. 10.1016/j.ajpe.2023.100615 37914030

