# Supplementary material for: A scoping review on the impact of versatile Digital Health innovations on pharmacy education
Source: Front Med (Lausanne). 2025 Oct 17;12:1577494. doi: 10.3389/fmed.2025.1577494 (PMC12575382; doi:10.3389/fmed.2025.1577494)
Supplement: Supplementary file 1 [file Table_1.docx]

| **Author, year** | **Study design** | **Population** | **Intervention** | **Comparison** | **Outcomes** |
| --- | --- | --- | --- | --- | --- |
| **Alsultan et al. (2024) [28]** | Cross-sectional study | Pharmacy students from five universities in Saudi Arabia | Integration of tele pharmacy education into the pharmacy curriculum | Traditional learning | Students' knowledge and perceptions of tele pharmacy education |
| **Hasan et al. (2024) [29]** | Cross-sectional study | Pharmacy students from the Middle East and North Africa (MENA) region | Exploration of attitudes towards AI integration in pharmacy education and practice | Focusing on students' concerns and influences on attitudes | Insight into ethical concerns and the need for comprehensive AI education in curricula |
| **Imad Mohammed et al. (2024) [30]** | Qualitative study using face-to-face interviews | Senior pharmacy students at two pharmacy colleges in Iraq | Use of artificial intelligence (AI), particularly ChatGPT, for academic purposes | Traditional study methods | Perceptions of the benefits and drawbacks of AI in enhancing or hindering academic skills |
| **Ali Sherazi et al. (2024) [33]** | Randomized crossover non-inferiority trial | German pharmacy students in their final semester | Telepharmacy inhaler technique training service | Face-to-face inhaler technique training | Student performance in demonstrating inhaler technique, confidence, and perceptions of tele pharmacy |
| **Alfian et al. (2023) [31]** | cross-sectional study | Pharmacy students in Indonesia | Assessment of knowledge, perception, and willingness to provide tele pharmacy services | Internal factors (e.g., smartphone proficiency, age, gender) | Insights into students' tele pharmacy knowledge, perceptions, and willingness to integrate tele pharmacy in future practice |

**Appendix 1** PICOs of selected studies on tele pharmacy, remote health services, and AI in education

**Keys**. **AI** (Artificial Intelligence), **MENA** (Middle East and North Africa), and **tele pharmacy** (remote delivery of pharmacy services).

| **Author, year** | **Study design** | **Population** | **Intervention** | **Comparison** | **Outcomes** |
| --- | --- | --- | --- | --- | --- |
| **Isamuddin et al. (2023) [11]** | Qualitative study | Undergraduate pharmacy students in Malaysia | Development of a tele pharmacy training module | Traditional study methods | Identification of essential components for tele pharmacy training to improve knowledge and skills |
| **Frenzel & Porter (2023) [34]** | Mixed-method research with post-surveys and thematic analysis | Second- and third-year pharmacy students at two pharmacy schools | Tele pharmacy and telehealth training module | Traditional study methods | Knowledge increase, attitudes, behavioral control, and intent to use telehealth |
| **Kharaba et al. (2023) [35]** | Randomized controlled head-to-head comparative assessment | Fourth-year pharmacy students at Al Ain University | Comparison between on-campus and virtual OSCE | On-campus OSCE vs. virtual OSCE | Student and examiner satisfaction, feasibility, and skill assessment efficacy |
| **Elnaem et al. (2022) [32]** | Cross-sectional survey | Senior pharmacy students in a Malaysian public university | Assessment of tele pharmacy knowledge, perceptions, and readiness | Internal analysis of responses | Levels of knowledge, perception, and readiness towards tele pharmacy |
| **Arman Rabbani et al (2021) [36]** | Descriptive report of a virtual training program | Final-year Bachelor of Pharmacy students in the UAE | Virtual experiential training across community and hospital pharmacy settings | Traditional in-person experiential training | Successful learning outcomes achieved, though not fully replacing in-person experience |

**Cont. Appendix 1** PICOs of selected studies on tele pharmacy, remote health services, and AI in education

**Keys. Tele pharmacy** (remote delivery of pharmacy services), **tele health** (healthcare services delivered remotely), and **OSCE** (Objective Structured Clinical Examination).
