# Supplementary material for: A scoping review on the impact of versatile Digital Health innovations on pharmacy education
Source: Front Med (Lausanne). 2025 Oct 17;12:1577494. doi: 10.3389/fmed.2025.1577494 (PMC12575382; doi:10.3389/fmed.2025.1577494)
Supplement: Supplementary file 2 [file Table_2.docx]

**Appendix 2** PICOs of selected studies on practical skill development and interactive learning through digital tools

| **Author, year** | **Study design** | **Population** | **Intervention** | **Comparison** | **Outcomes** |
| --- | --- | --- | --- | --- | --- |
| **Pihl et al. (2021) [2]** | Qualitative study | Swedish pharmacy students | Education on tele pharmacy and patient communication at a distance | None explicitly mentioned; indirect comparison to traditional face-to-face communication | Students' preparations for tele pharmacy; their views on the adequacy of current education |
| **Tai et al (2020) [43]** | Cohort study | First-year pharmacy students in a self-care therapeutics course | Virtual simulation using MyDispense in self-care therapeutics | Traditional course format without virtual simulation | Frequency of interactions during introductory pharmacy practice experience ,student confidence, preceptor-reported student performance |
| **Ameri et al. (2020) [37]** | Cross-sectional study | Pharmacy students in Iran | Usage of a mobile-based educational application | Traditional learning methods | Behavioral intention to use and long-term acceptance of the LabSafety app |
| **Rahman et al. (2020) [44]** | Case study | Pharmacy students in Malaysia | Remote experiential learning in community pharmacy | Traditional, on-site community pharmacy experiential learning | Skill acquisition in dispensing, OTC consultations, and overall pharmacy practice |
| **Tomevska Ilievska et al. (2019) [38]** | Qualitative study with educational assessments | Undergraduate pharmacy students | Interactive teaching models, including group discussions, tutorials, and problem-solving activities | Traditional teaching methods | Increased engagement, active learning, and long-term knowledge retention |

**Keys**. **Tele pharmacy** (remote delivery of pharmacy services), **OTC** (Over the Counter), and **virtual simulation** (use of digital simulations for practical training).

| **Author, year** | **Study design** | **Population** | **Intervention** | **Comparison** | **Outcomes** |
| --- | --- | --- | --- | --- | --- |
| **Ros Castellar et al. (2024) [39]** | Training evaluation and satisfaction survey | Pharmacy technicians in a tertiary hospital | Compounding training program using digital e-learning and simulation | Traditional study methods | Skill acquisition, satisfaction, and qualification in sterile and non-sterile compounding |
| **Kurniawan et al. (2023) [25]** | Quasi-experimental study | Pharmacy students from Indonesia | Use of Android-based augmented reality (AR) models for learning about diabetes mellitus drug information | Traditional learning methods without AR | Improvement in pharmacy students' knowledge, engagement, and satisfaction regarding diabetes mellitus drugs |
| **Obarcanin et al. (2022) [40]** | descriptive study | Final-year pharmacy students | m-Health course focusing on digital diabetes apps | Traditional learning methods | Improved digital skills for managing diabetes in clinical pharmacy |
| **Oktianti et al. (2022) [8]** | Pre-test/post-test training intervention | Pharmacists in Indonesia | Training in digital media use for digital pharmacy | Knowledge levels pre- and post-training | Increased knowledge of digital pharmacy and medication therapy management |
| **Nounou et al. (2022) [41]** | Cross-over study design conducted across two cohorts | First-year pharmacy students in a pharmaceutical compounding laboratory course | Implementation of a mobile-based augmented reality (AR) application (AmplifiedRx app) in pharmaceutical labs | AR application use versus traditional lab methods without AR | Enhanced student engagement, improved understanding and confidence in laboratory skills, and high acceptability of the AR technology |
| **kapp et al. (2022) [42]** | Pilot study with pre- and post-surveys | Pharmacy students in a university laboratory course | Augmented reality (AR) environment using smart glasses and mobile devices | Traditional laboratory teaching methods without AR | Student perceptions of AR as a learning aid, user experience, self-efficacy, and anxiety towards technology |

**Cont. Appendix 2** PICOs of selected studies on practical skill development and interactive learning through digital tools

**Keys**. **AR** (Augmented Reality), **mHealth** (mobile health applications).
