# Supplementary material for: A scoping review on the impact of versatile Digital Health innovations on pharmacy education
Source: Front Med (Lausanne). 2025 Oct 17;12:1577494. doi: 10.3389/fmed.2025.1577494 (PMC12575382; doi:10.3389/fmed.2025.1577494)
Supplement: Supplementary file 3 [file Table_3.docx]

| **Author, year** | **Study design** | **Population** | **Intervention** | **Comparison** | **Outcomes** |
| --- | --- | --- | --- | --- | --- |
| **Busch et al. (2024) [45]** | cross-sectional study | International pharmacy students | Survey on attitudes towards AI in pharmacy education | Traditional learning methods | Attitudes and preparedness toward AI in pharmacy |
| **Anderson et al (2024) [46]** | cross-sectional study | Pharmacy students in a U.S. PharmD program | Use of ChatGPT for personal, academic, and clinical purposes | Traditional learning methods | Patterns of ChatGPT usage and perceptions regarding its inclusion in curricula |
| **Naguib et al (2023) [47]** | Prospective, mixed-methods study | Fifth-year pharmacy students at Egypt | Virtual training on clinical pharmacy using the Virtual Faculty of Pharmacy Cairo University platform | Comparison between pretest and posttest scores pre- and post-virtual training | Improved academic performance and high student satisfaction |
| **Sodnom et al (2021) [49]** | Mixed-methods study including surveys and statistical analysis | Undergraduate students | E-learning during the COVID-19 pandemic | Classroom-based traditional learning methods | Evaluation of learning effectiveness, student satisfaction, and challenges of e-learning compared to classroom training |

**Appendix 3** PICOs of selected studies on student and faculty perceptions, attitudes, and challenges in adopting digital health.

**Keys.** **AI** (Artificial Intelligence), **ChatGPT** (a language model used for academic and clinical purposes), **U.S** (United State), **e-learning** (online learning).

| **Author, year** | **Study design** | **Population** | **Intervention** | **Comparison** | **Outcomes** |
| --- | --- | --- | --- | --- | --- |
| **Newsom et al. (2021) [48]** | cross-sectional study | First- and third-year pharmacy students at Mercer University | Digital poster presentations | Printed poster presentations | Students’ perceptions of learning tools and preferences |
| **Bautista et al ( 2020) [50]** | Descriptive report on a clinical rotation | Pharmacy and medical students | Interprofessional telehealth rotation for outreach to vulnerable patients | Traditional in-person rotations were not directly compared | Enhanced interprofessional communication, understanding of roles, and telehealth skills |
| **Kelm & Bush (2020) [51]** | Descriptive report on program development | Pharmacy technician students | Pharmacy technician training program using digital content | Traditional learning methods | Skills development, certification readiness, and retention |
| **Jenkins et al. (2020) [9]** | observational study | Pharmacy students and residency program directors | Examine the effectiveness and perception of PhORCAS | Traditional methods | The critical role of tailored educational tools and supportive learning environments in preparing pharmacy students for successful careers |

**Cont. Appendix 3** PICOs of selected studies on student and faculty perceptions, attitudes, and challenges in adopting digital health

**Keys. PhORCAS** (Pharmacy Online Residency Centralized Application Service), and **tele health** (remote healthcare services).
